# Influence of Different Cavity Disinfection Protocols on Adhesion at the Resin Composite–Dentin Interface

**DOI:** 10.3390/polym18091011

**Published:** 2026-04-22

**Authors:** Soner Sismanoglu, Zeynep Hale Keles, Vasfiye Işık

**Affiliations:** 1 Department of Restorative Dentistry, Faculty of Dentistry, Istanbul University-Cerrahpasa, 34093 Istanbul, Turkey; 2 Department of Restorative Dentistry, Faculty of Dentistry, Istanbul Atlas University, Atlas Vadi Kampüsü, Anadolu Cd. No: 40 Kağıthane, 34408 Istanbul, Turkey; 3 Department of Endodontics, Faculty of Dentistry, Istanbul University-Cerrahpasa, 34093 Istanbul, Turkey; vasfiye.isik@iuc.edu.tr

**Keywords:** cavity disinfectant, chlorhexidine, hypochlorous acid, microtensile bond strength, universal adhesive

## Abstract

This study evaluated the effects of four cavity disinfection protocols on microtensile bond strength (µTBS) and failure mode of dentin bonded with a universal adhesive in self-etch mode. Sixty human third molars were assigned to five groups (*n* = 12): Control (Clearfil S3 Bond Universal), Clearfil SE Protect Bond (CPB, MDPB-containing), 2% chlorhexidine (CHX), 5.25% sodium hypochlorite (NaOCl), and 200 ppm hypochlorous acid (HOCl). After disinfectant application and bonding, composite build-ups were sectioned into beams (≈0.9 mm^2^) and tested as immediate (24 h) and thermocycled (10,000 cycles) subgroups. Data were analyzed using two-way ANOVA, Tukey HSD, and chi-square/Fisher’s exact tests (α = 0.05). At 24 h, NaOCl and CHX produced significantly lower µTBS than the control, HOCl, and CPB groups (*p* < 0.05). After thermocycling, Control, CPB, and NaOCl declined significantly, while CHX remained stable (*p* = 0.960) and HOCl showed non-significant reduction (*p* = 0.086). NaOCl yielded the highest adhesive failure rate and lowest bond strength. CHX reduced initial µTBS but maintained stability. HOCl and CPB produced values comparable to controls, though HOCl was more aging-susceptible. MDPB-containing adhesives may preserve bond durability while providing disinfection.

## 1. Introduction

Achieving a durable bond between resin-based restorative materials and dentin remains one of the central challenges of adhesive dentistry. The longevity of composite restorations is contingent not only on the mechanical and chemical properties of the adhesive system, but also on the condition of the underlying dentin substrate at the time of bonding. Cavity preparation, even when performed meticulously, cannot guarantee a bacteria-free surface; histological and bacteriological evidence consistently demonstrates that viable cariogenic microorganisms can persist within dentinal tubules, the smear layer, and at the dentin-enamel junction following caries excavation [1]. These residual bacteria, if left sealed beneath a restoration, may remain viable for extended periods and contribute to secondary caries, pulpal inflammation, and ultimately restoration failure [2]. Beyond bacterial risk, the hybrid layer itself is susceptible to time-dependent degradation: collagenolytic host enzymes, primarily matrix metalloproteinases (MMP)-2, -8, and -9, are activated during dentin demineralization and progressively degrade incompletely resin-infiltrated collagen fibrils, compromising interfacial integrity over time [3]. The application of a cavity disinfectant prior to bonding has therefore been proposed as a complementary measure to mechanical caries removal, ideally one capable of both eliminating residual bacteria and preserving (or even enhancing) the long-term stability of the adhesive interface.

Among the agents evaluated for this purpose, 2% chlorhexidine digluconate (CHX) has attracted the most sustained attention. Its broad-spectrum antimicrobial activity is well established, but the interest in CHX has extended well beyond simple bacterial reduction. CHX is a potent inhibitor of MMPs and cysteine cathepsins, and its application to acid-etched or self-etched dentin has been shown to significantly slow the degradation of the hybrid layer [4,5]. A meta-analysis of 21 studies confirmed that while CHX pretreatment has no significant effect on immediate bond strength values, bond strengths in the CHX-treated group were significantly higher than controls after 6, 12, and 24 months of aging [6]. Notably, Breschi et al. [7] demonstrated in an accelerated aging model that CHX remains within the hybrid layer after 10 years, retaining measurable MMP-inhibitory activity and preserving the structural integrity of the collagen network. Despite this promising durability profile, the immediate bond strength effects of CHX in conjunction with contemporary universal adhesives in self-etch mode remain inconsistent across studies, warranting further investigation.

Sodium hypochlorite (NaOCl) represents an entirely different approach to cavity disinfection, one derived from its established role in endodontic irrigation. At concentrations of 5.25%, NaOCl exerts potent oxidative and proteolytic activity capable of virtually eliminating residual bacteria from cavity surfaces. However, the same oxidizing mechanism that drives its antimicrobial efficacy has been shown to adversely affect the dentin-adhesive interface through multiple pathways: dissolution and denaturation of collagen fibrils impairs hybrid layer formation, while residual protein chloramine-derived radicals generated during NaOCl application can cause premature termination of free-radical polymerization chains, leading to incomplete adhesive conversion at the dentin surface [8,9]. This oxidative interference is particularly problematic for self-etching systems, which rely on intact residual hydroxyapatite for chemical bonding and cannot effectively rinse away NaOCl by-products as etch-and-rinse protocols can [10]. These findings have prompted many investigators to caution against the routine use of NaOCl as a cavity disinfectant when resin bonding is to follow.

Hypochlorous acid (HOCl) has more recently emerged as a biologically derived oxidizing agent with a potentially more favorable profile for use prior to adhesive bonding. Naturally produced by neutrophils as part of the innate immune response, HOCl combines broad-spectrum antimicrobial activity with substantially greater biocompatibility than NaOCl at the concentrations relevant to clinical use [11,12]. Studies examining HOCl at concentrations between 50 and 200 ppm have demonstrated that smear layer deproteinization can be achieved without the degree of collagen damage associated with hypochlorite, and critically, without the residual oxidant activity that impairs adhesive polymerization [13]. Recent work has shown that brief HOCl application can increase the immediate µTBS of one-step self-etch adhesives to dentin and improve bonding durability after thermocycling when combined with appropriate adjuncts [14]. Nevertheless, the evidence base for HOCl as a clinical cavity disinfectant remains limited, and its interaction with MDP-containing universal adhesive systems at clinically relevant concentrations and application times has not yet been systematically characterized.

An alternative to sequential disinfection-then-bonding is offered by adhesive systems that incorporate antibacterial functionality directly into their formulation. Clearfil SE Protect Bond (Kuraray Noritake Dental, Tokyo, Japan), a two-step self-etching adhesive, contains the quaternary ammonium monomer 12-methacryloyloxydodecylpyridinium bromide (MDPB) in its primer component, which exerts a contact-killing effect against a broad range of oral pathogens during bonding, effectively integrating disinfection and adhesion into a single clinical step [15]. After polymerization, the MDPB monomer is immobilized within the adhesive layer, ensuring the antibacterial effect is retained without compromising mechanical properties or bond durability [16]. Universal adhesives, on the other hand, have grown to dominate clinical practice due to their compatibility with multiple etching strategies. When applied in self-etch mode, the residual hydroxyapatite preserved by partial demineralization serves as a substrate for chemical bonding via functional monomers such as 10-MDP, which forms stable ionic bonds with calcium ions to generate a self-assembled MDP-Ca nano-layer at the adhesive interface, a mechanism closely linked to long-term bond durability [17,18]. How these different disinfection strategies interact with this bonding mechanism, particularly under thermocycling stress simulating the oral aging environment, remains incompletely defined.

Although CHX, NaOCl, and MDPB-containing adhesives have been individually evaluated in various bonding contexts, the effect of HOCl as a cavity disinfectant prior to bonding with an MDP-containing universal adhesive in self-etch mode has not been investigated. Moreover, no study to date has directly compared these mechanistically distinct disinfection strategies within a single standardized experimental design. The purpose of this in vitro study was therefore to evaluate the effects of four cavity disinfection protocols (2% CHX, 5.25% NaOCl, 200 ppm HOCl, and the MDPB-containing self-etching adhesive system Clearfil SE Protect Bond) on the immediate and thermocycled microtensile bond strength (µTBS) of dentin bonded with a 10-MDP-containing universal adhesive in self-etch mode, relative to an untreated control. The null hypotheses tested were: (1) the type of cavity disinfectant applied prior to bonding would have no significant effect on µTBS values; (2) thermocycling aging would not significantly reduce µTBS values; and (3) neither disinfectant type nor aging would influence the distribution of failure modes at the adhesive interface.

## 2. Materials and Methods

### 2.1. Study Design and Specimen Preparation

**Figure 1:**
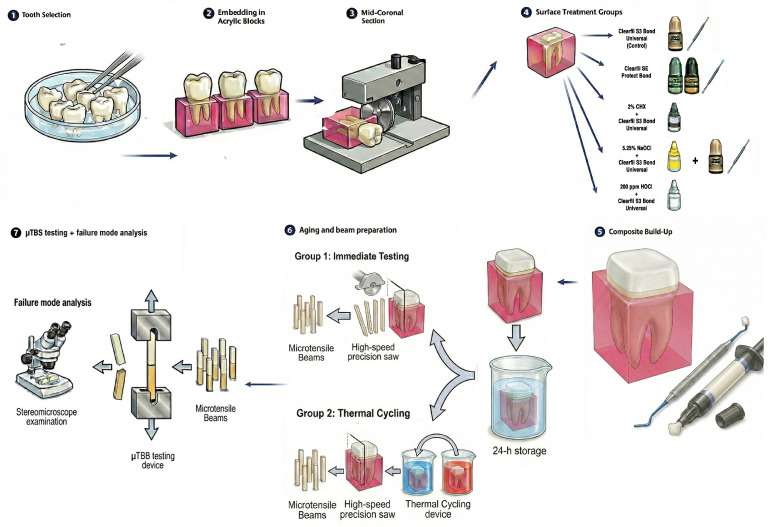
Schematic illustration of the experimental workflow. Sixty caries-free third molars were embedded in acrylic resin, and mid-coronal dentin was exposed. Specimens were randomly assigned to five surface treatment groups: Control (Clearfil S3 Bond Universal), Clearfil SE Protect Bond (CPB), 2% chlorhexidine (CHX), 5.25% sodium hypochlorite (NaOCl), or 200 ppm hypochlorous acid (HOCl), followed by adhesive application (Clearfil S3 Bond Universal) and composite build-up. After 24 h storage, bonded teeth were sectioned into beams (≈0.9 mm^2^) and divided into immediate testing and thermocycled (10,000 cycles) groups. Microtensile bond strength (µTBS) was measured, and failure modes were analyzed under stereomicroscope.

This in vitro study was approved by the Altınbaş University Clinical Research Ethics Committee (Decision No: 2022/144). The study was conducted in accordance with the Declaration of Helsinki. Written informed consent was obtained from all patients prior to tooth extraction, explicitly allowing the use of their extracted teeth for research purposes. The experimental design is illustrated schematically in Figure 1. The sample size was determined based on an a priori power analysis using data from previous studies evaluating the effects of HOCl and NaOCl pretreatment on µTBS of self-etch adhesives to dentin [13,14], with a within-group standard deviation of approximately 5.0 MPa and a large effect size (Cohen’s f = 0.82). For a two-way ANOVA (five disinfectant groups × two aging conditions) with 6 teeth per cell (total N = 60 teeth) at α = 0.05, the calculated power for detecting the main effect of disinfectant group exceeded 0.99 (G*Power 3.1; Heinrich Heine University Düsseldorf, Düsseldorf, Germany).

Sixty caries-free human third molars extracted for clinical indications were used in this study. Teeth were stored in 0.5% chloramine-T (Merck, Darmstadt, Germany) solution at 4 °C and used within one month of extraction. Calculus and soft tissue remnants were removed with a periodontal scaler. The crowns were embedded in self-curing acrylic resin (Imicryl; Konya, Turkey) with the occlusal surfaces oriented parallel to the floor. Mid-coronal dentin sections were obtained using a precision cutting device (IsoMet High Speed Pro; Buehler Ltd., Lake Bluff, IL, USA). The exposed dentin surfaces were subsequently polished with 600-grit silicon carbide (SiC) paper to create a standardized smear layer. The absence of enamel on the bonding surface was confirmed under a stereomicroscope at ×10 magnification.

### 2.2. Experimental Groups

The sixty specimens were randomly allocated to five experimental groups (*n* = 12 per group) according to the cavity disinfectant applied prior to adhesive bonding using a computer-generated randomization sequence (Random.org, Dublin, Ireland). Operator blinding was not feasible due to the visibly different application protocols of the disinfectant agents; however, bond strength testing and failure mode analysis were performed by an examiner blinded to group allocation. The experimental groups and bonding protocols are summarized in Table 1.

Group 1, Control: The dentin surface was rinsed with distilled water for 30 s and gently air-dried, followed by application of Clearfil S3 Bond Universal in self-etch mode.

Group 2, Clearfil SE Protect Bond (CPB): Clearfil SE Protect Primer (Kuraray Noritake Dental, Tokyo, Japan), which contains the antibacterial monomer 12-methacryloyloxydodecylpyridinium bromide (MDPB), was applied to the dentin surface and left undisturbed for 20 s, followed by gentle air-drying for 5 s. CPB was then applied, spread with mild air, and light-cured for 10 s. This group served as a positive control for antibacterial adhesive bonding, as the MDPB-containing self-etching system simultaneously provides cavity disinfection and adhesive bonding without the use of a separate disinfectant agent.

Group 3, Chlorhexidine (CHX): 2% chlorhexidine digluconate solution (Consepsis, Ultradent, South Jordan, UT, USA) was applied to the dentin surface using a microbrush for 60 s. Excess solution was removed by blot-drying with a pellet, leaving the surface moist, followed by application of Clearfil S3 Bond Universal in self-etch mode.

Group 4, Sodium Hypochlorite (NaOCl): 5.25% sodium hypochlorite solution (Chloraxid; Cerkamed, Skoczów, Poland) was applied to the dentin surface for 30 s, followed by thorough water rinsing for 30 s and gentle air-drying, followed by application of Clearfil S3 Bond Universal in self-etch mode.

Group 5, Hypochlorous Acid (HOCl): 200 ppm hypochlorous acid solution (Superox; Ankara, Turkey) was applied to the dentin surface using a microbrush for 30 s. Excess solution was removed by blot-drying with a pellet, leaving the surface moist, followed by application of Clearfil S3 Bond Universal in self-etch mode.

### 2.3. Adhesive Application and Composite Resin Build-Up

For Groups 1, 3, 4, and 5, Clearfil S3 Bond Universal (Kuraray Noritake Dental, Tokyo, Japan) was applied in self-etch mode according to the manufacturer’s recommendations. The adhesive was applied with a microbrush and actively agitated for 20 s, followed by gentle air-thinning for 5 s and light-curing for 10 s. For Group 2, the CPB adhesive system was applied as described in experimental groups. All light-curing procedures were performed using an LED curing unit (VALO Grand; Ultradent, South Jordan, UT, USA) at a standard output of 1000 mW/cm^2^.

A nanofill resin composite (Filtek Ultimate Universal; 3M ESPE Dental Products, St. Paul, MN, USA) was applied incrementally in 2 mm thick layers to a total height of 5 mm. Each increment was light-cured for 20 s. All bonded specimens were stored in artificial saliva at 37 °C for 24 h prior to sectioning.

### 2.4. Specimen Sectioning and Aging Protocol

Each bonded tooth was longitudinally sectioned in both x- and y-directions across the adhesive interface using a precision cutting machine (IsoMet High Speed Pro; Buehler Ltd., Lake Bluff, IL, USA) under water cooling to obtain resin–dentin beams with a cross-sectional area of approximately 0.9 mm^2^. Peripheral beams were discarded. The remaining beams from each tooth were randomly and equally assigned to two subgroups based on the aging condition (*n* = 6 teeth per group per time point). For the immediate subgroup, beams were stored in artificial saliva at 37 °C for 24 h and then subjected to µTBS testing. For the thermocycled subgroup, beams were subjected to 10,000 thermal cycles between 5 °C and 55 °C, with a dwell time of 30 s and a transfer time of 10 s (MOD Thermocycler; Esetron Smart Robotechnologies, Ankara, Turkey). During the thermocycling process, specimens were immersed in artificial saliva. Following thermocycling, the beams were stored in artificial saliva at 37 °C for 24 h prior to µTBS testing.

### 2.5. Microtensile Bond Strength Testing

The cross-sectional dimensions of each beam were measured with a digital caliper (ABS Digimatic; Mitutoyo Corp., Tokyo, Japan) and recorded in mm^2^. Each beam was attached to a microtensile testing device (MOD Tester; Esetron Smart Robotechnologies, Ankara, Turkey) using a cyanoacrylate adhesive (Two-Component Fast-Setting Adhesive; Polisan, Istanbul, Turkey) and subjected to tensile force at a crosshead speed of 0.5 mm/min until failure. The µTBS was calculated by dividing the maximum load at failure (N) by the cross-sectional area (mm^2^) and expressed in MPa. No pre-test failures occurred during sectioning or handling.

### 2.6. Failure Mode Analysis

The fracture surfaces of all tested specimens were examined under a stereomicroscope (SMZ745T; Nikon, Tokyo, Japan) at ×40 magnification. Failure modes were categorized as: (i) adhesive, failure at the resin–dentin interface; (ii) cohesive in dentin, failure within the dentin substrate; (iii) cohesive in composite, failure within the resin composite; and (iv) mixed, failure involving both adhesive and cohesive components. All evaluations were performed by a single calibrated examiner who was blinded to group allocation.

### 2.7. Statistical Analysis

For µTBS analysis, the statistical unit was the tooth; mean bond strength values per tooth were calculated to avoid pseudo-replication from multiple beams obtained from the same specimen. The normality of data distribution was assessed using the Shapiro–Wilk test, and variance homogeneity was evaluated using the Levene test. As assumptions for parametric testing were fulfilled, a two-way analysis of variance (ANOVA) was applied to evaluate the effects of disinfectant group, aging condition (immediate vs. thermocycled), and their interaction on µTBS values. Post hoc pairwise comparisons were performed using the Tukey HSD test. Failure mode distributions among groups were compared using the chi-square test, and within-group differences between immediate and thermocycled conditions were analyzed using Fisher’s exact test. Statistical analyses were performed using IBM SPSS Statistics (version 31; IBM Corp., Armonk, NY, USA). The level of significance was set at α = 0.05 for all analyses.

## 3. Results

### 3.1. Microtensile Bond Strength

**Figure 2:**
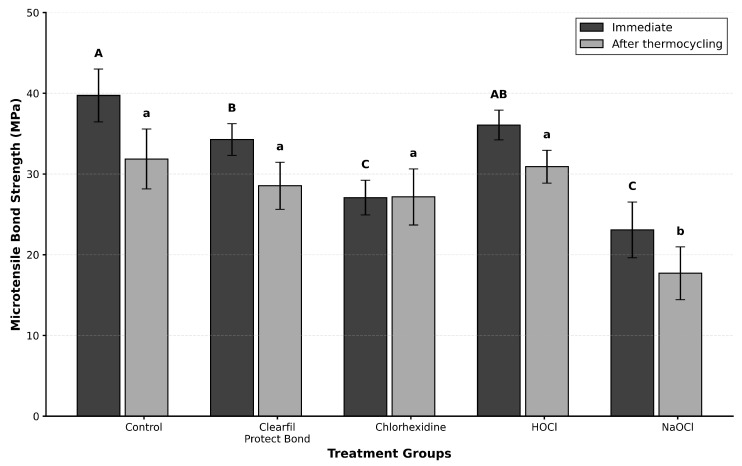
Microtensile bond strength (µTBS) values of the experimental groups measured immediately (24 h) and after thermocycling (10,000 cycles). Bars represent mean µTBS values per tooth (statistical unit: tooth), and error bars indicate standard deviations. Different uppercase letters indicate statistically significant differences among groups at the immediate evaluation, whereas different lowercase letters indicate significant differences among groups after thermocycling (*p* < 0.05). HOCl, hypochlorous acid; NaOCl, sodium hypochlorite.

The mean µTBS values for all experimental groups at both time points are presented in Table 2 and illustrated in Figure 2. Two-way ANOVA revealed significant main effects of group (F(4, 50) = 53.03, *p* < 0.001) and time (F(1, 50) = 41.44, *p* < 0.001), as well as a significant group × time interaction (F(4, 50) = 3.10, *p* = 0.024), indicating that the effect of thermocycling on bond strength differed among the groups.

At the immediate evaluation, the control group exhibited the highest µTBS (39.7 ± 3.3 MPa), followed by the HOCl (36.1 ± 1.8 MPa), CPB (34.3 ± 2.0 MPa), chlorhexidine (27.1 ± 2.2 MPa), and NaOCl (23.1 ± 3.5 MPa) groups. Post hoc analysis demonstrated that the control group yielded significantly higher bond strength than the CPB group (*p* = 0.011), whereas no significant difference was observed between the HOCl and CPB groups (*p* = 0.759). Both the control and HOCl groups were significantly superior to the chlorhexidine and NaOCl groups (*p* < 0.001 for all comparisons). No significant difference was observed between the control and HOCl groups (*p* = 0.145). The NaOCl group recorded the lowest immediate bond strength, which was significantly lower than all other groups (*p* < 0.001). No significant difference was observed between the chlorhexidine and NaOCl groups (*p* = 0.087).

After 10,000 thermocycles, bond strength values decreased significantly in the control (31.9 ± 3.7 MPa, *p* = 0.001), CPB (28.5 ± 2.9 MPa, *p* = 0.037), and NaOCl (17.7 ± 3.3 MPa, *p* = 0.002) groups. The HOCl group exhibited a numerically substantial reduction (30.9 ± 2.0 MPa) that did not reach statistical significance (*p* = 0.086). In contrast, the chlorhexidine group demonstrated stable bond strength values following thermocycling (27.2 ± 3.5 MPa, *p* = 0.960). At this time point, NaOCl yielded significantly lower bond strength than all other groups (*p* < 0.001), whereas no significant differences were observed among the remaining four groups (*p* > 0.05).

### 3.2. Failure Mode Analysis

The distribution of failure modes for all groups is summarized in Table 3. Chi-square analysis revealed a significant difference in failure mode distribution among the groups at the immediate evaluation (χ^2^(12) = 40.11, *p* < 0.001), whereas no significant intergroup difference was detected after thermocycling (χ^2^(12) = 15.56, *p* = 0.212).

At immediate evaluation, mixed-type failure was the predominant mode in the control (72.4%) and HOCl (56.9%) groups, while adhesive failure was most frequent in the NaOCl (52.6%) and chlorhexidine (38.7%) groups. After thermocycling, the proportion of adhesive failures increased across all groups, with statistically significant increases observed in the control (10.3% to 36.9%, *p* = 0.001) and HOCl (17.2% to 42.6%, *p* = 0.004) groups. The chlorhexidine group showed a negligible change in adhesive failure proportion (38.7% to 39.3%, *p* = 1.000), consistent with its stable bond strength values over time. The NaOCl group recorded the highest adhesive failure percentage at both time points (52.6% and 61.3%, respectively), although the increase did not reach statistical significance (*p* = 0.360). Overall, groups with lower mean µTBS values tended to exhibit higher proportions of adhesive failure, indicating that bond strength and failure mode outcomes were consistent in reflecting the degree of interfacial integrity across the experimental conditions.

## 4. Discussion

This study evaluated the effects of four cavity disinfection protocols (2% CHX, 5.25% NaOCl, 200 ppm HOCl, and the MDPB-containing adhesive system [CPB]) on the immediate (24 h) and thermocycled (10,000 cycles) µTBS of dentin bonded with a 10-MDP-containing universal adhesive in self-etch mode. At 24 h, NaOCl and CHX produced significantly lower µTBS than the control, HOCl, and CPB groups; notably, CHX and NaOCl were statistically indistinguishable from each other, both falling into the lowest-performing subgroup. After thermocycling, µTBS decreased significantly in the control, CPB, and NaOCl groups; CHX remained virtually unchanged (27.1 to 27.2 MPa, *p* = 0.960), while HOCl exhibited a numerically pronounced reduction (36.1 to 30.9 MPa) that nonetheless did not reach statistical significance (*p* = 0.086). The control group showed the greatest absolute numerical reduction in µTBS, while NaOCl showed the greatest proportional decrease; no statistically significant differences were observed among the control, CPB, HOCl, and CHX groups after thermocycling. Failure mode distribution shifted toward adhesive fractures with thermocycling in all groups, most markedly in the NaOCl group. Accordingly, the first null hypothesis (that disinfectant type would not affect µTBS) was partially rejected (due to the NaOCl and CHX groups’ significantly lower 24 h values and the between-group differences following thermocycling). The second null hypothesis (that thermocycling would not affect µTBS) was partially rejected: a statistically significant decrease was observed in the control, CPB, and NaOCl groups, while CHX remained stable (*p* = 0.960) and the HOCl group showed a numerically substantial but statistically non-significant reduction (*p* = 0.086). The third null hypothesis (that neither disinfectant type nor aging would influence failure mode distribution) was also rejected.

The finding that 2% CHX application prior to Clearfil S3 Bond Universal in self-etch mode produced significantly lower immediate µTBS compared to the control, HOCl, and CPB groups warrants careful consideration. While a systematic review/meta-analysis including 21 CHX studies found no significant difference between CHX-treated and control groups at baseline [6], the present finding of reduced 24 h bond strength in the CHX group may reflect the particular chemistry of this universal adhesive in self-etch mode, in which calcium ions released during the self-etch process may chelate CHX and precipitate insoluble calcium-CHX complexes at the bonding surface, potentially interfering with monomer infiltration and polymerization [19]. The neutral effect of CHX at 24 h reported in prior studies is generally attributed to its rewetting capacity, which helps maintain the hydrated state of the demineralized collagen network [4]; however, this compensatory mechanism may be insufficient in the presence of calcium chelation. A more clinically meaningful observation in the present study is the stability of the CHX group after thermocycling (27.1 to 27.2 MPa, *p* = 0.960)—the only group to show no statistically significant change—consistent with CHX’s established role as an MMP inhibitor. The well-documented mechanism involves the non-competitive inhibition of endogenous dentin MMPs (-2, -8, -9) and cysteine cathepsins that are activated by the acidic monomer during self-etch bonding, thereby slowing the proteolytic degradation of incompletely resin-encapsulated collagen fibrils at the base of the hybrid layer [5,20]. Thus, while CHX compromised immediate bond strength in the current experimental model, it demonstrated stable bond strength after thermocycling—a trade-off with important implications for clinical interpretation.

Among the disinfectant-treated groups, the CPB group achieved the highest µTBS both at 24 h and after thermocycling, and was the only disinfectant group to maintain values statistically comparable to the untreated control at both time points. This outcome is consistent with the potential of MDPB-containing self-etching adhesives as an integrated approach to both disinfection and bonding. The antibacterial monomer MDPB in the Clearfil SE Protect primer exerts direct contact-killing against oral pathogens during bonding without any adverse effect on the adhesive interface, as the monomer becomes immobilized upon polymerization [15,16]. The relatively favorable bond stability after thermocycling of the CPB group can plausibly be attributed to the two-step self-etching protocol of Clearfil SE Protect, which employs a separate primer step that achieves more thorough monomer infiltration and creates a more clearly defined hybrid layer compared to one-step universal adhesives [21]. This additional primer step may produce a more complete encapsulation of exposed collagen fibrils, leaving fewer vulnerable sites for MMP-mediated degradation. Furthermore, the chemical bonding contribution of 10-MDP in the bond component of Clearfil SE Protect, which also forms the stable MDP-Ca nano-layer at the adhesive interface, likely reinforces bond durability under thermocycling stress [18].

The significantly reduced 24 h µTBS in the NaOCl group confirms the adverse effect of 5.25% NaOCl on the dentin-adhesive interface and provides clear grounds for rejecting the first null hypothesis. This reduction is consistent with the multiple mechanisms through which NaOCl compromises bonding: denaturation and dissolution of collagen fibrils impairs hybrid layer formation and reduces the collagen network available for micromechanical interlocking, while residual protein chloramine-derived radicals generated by the oxidant interfere with free-radical polymerization of the adhesive resin, leading to incomplete monomer conversion at the dentin surface [8,9]. Self-etching adhesives are particularly vulnerable to this oxidative interference compared to etch-and-rinse systems, as the latter can partially remove NaOCl residues along with the rinsing step, whereas in self-etch mode the oxidant remains in intimate contact with the bonding substrate [10]. The further deterioration of the NaOCl group after thermocycling and the marked shift toward adhesive failure modes, with adhesive fractures rising to 61.3% of specimens after 10,000 thermocycles, collectively suggest a compromised interface from the outset, one that is poorly equipped to withstand hygrothermal cycling stress. These findings corroborate the recommendation that NaOCl, despite its potent antimicrobial efficacy, should not be used as a routine cavity disinfectant preceding adhesive bonding without antioxidant neutralization [8]. Antioxidant agents have been shown to recover bond strength to NaOCl-treated dentin [22,23], and HOCl at lower concentrations does not impair immediate µTBS of self-etch adhesives, although its long-term bonding durability may require adjunctive agents [24]. The overall shift toward adhesive failure modes following thermocycling across all groups, most markedly in the NaOCl group, is consistent with progressive degradation at the adhesive-dentin interface [3] and supports the validity of thermocycling as an aging model for comparative bonding studies.

The HOCl group presented an interesting and somewhat nuanced pattern. At 24 h, µTBS values were statistically comparable to those of the control group, suggesting that the 200 ppm HOCl concentration used in this study did not acutely compromise adhesive monomer diffusion or polymerization. This is consistent with previous reports indicating that low-concentration HOCl solutions do not significantly alter dentin surface pH or cause the degree of collagen denaturation associated with NaOCl [13]. However, the HOCl group exhibited a marked decrease following thermocycling—a substantial reduction (36.1 to 30.9 MPa, approximately 14%)—with µTBS in the HOCl group after 10,000 thermocycles falling below that of the control group, though without reaching statistical significance relative to it. This pattern suggests that while HOCl does not acutely interfere with bonding, it may alter the dentin substrate in ways that reduce long-term interfacial resistance. One plausible explanation involves the smear layer deproteinization mechanism of HOCl: by partially depleting the organic matrix of the smear layer prior to self-etch primer application, the surface chemistry presented to the adhesive monomer may differ from that of unmodified dentin, potentially reducing the density of residual hydroxyapatite available for 10-MDP chemical bonding and thus weakening the ionic component of adhesion [14]. Consistent with this interpretation, Sanon et al. showed that HOCl pretreatment of smear-layer-covered dentin increased the immediate µTBS of one-step self-etch adhesives; however, HOCl-treated dentin also exhibited a significant thermocycling-induced µTBS decrease unless an additional sulfinate activator was applied post-deproteinization [24]. The failure mode distribution in the HOCl group at 24 h was notable for its high proportion of mixed failures (56.9%) combined with a relatively low adhesive failure rate (17.2%), a pattern consistent with a substrate that supports adequate interfacial engagement at baseline but may be susceptible to preferential failure at the hybrid layer under aging stress. Further investigation of HOCl concentrations, application times, and the potential benefit of adjunctive agents such as sulfinate activators in this context appears warranted. Given the limited evidence currently available, HOCl should be considered an experimental cavity disinfectant that requires further clinical validation, particularly regarding optimal concentration, application time, and potential need for adjunctive agents, before routine clinical recommendation.

From a clinical perspective, these findings suggest that the choice of cavity disinfectant should be guided not only by antimicrobial efficacy but also by compatibility with the adhesive system employed. When using MDP-based universal adhesives in self-etch mode, clinicians should be aware that NaOCl pretreatment without antioxidant neutralization may significantly compromise bond durability. CHX, despite reducing immediate bond strength, maintained stable values after thermocycling, which may be relevant in clinical scenarios where long-term restoration longevity is prioritized over initial bond performance. HOCl at 200 ppm produced immediate bond values comparable to the untreated control but showed a trend toward reduction after aging, suggesting that its clinical use requires further protocol optimization before routine recommendation. MDPB-containing adhesive systems such as Clearfil SE Protect offer the practical advantage of combining disinfection and bonding in a single clinical step without compromising bond durability. In practical terms, these results may inform a scenario-based approach to disinfectant selection: in deep cavities where residual bacteria are a primary concern and long-term bond stability is critical, CHX pretreatment may be preferred despite its initial bond strength reduction, given its documented MMP-inhibitory effect and the thermocycling stability observed in this study. In contrast, when preservation of immediate bond strength is the priority, such as in large direct restorations where early interfacial integrity is essential, HOCl or MDPB-containing systems may be more appropriate. NaOCl, while effective as an antimicrobial agent, should be reserved for situations where antioxidant neutralization can be performed prior to bonding, or where its use is specifically indicated for endodontic reasons rather than as a routine cavity disinfectant.

The present study has several limitations that should be considered when interpreting its findings. The experimental substrate consisted of sound coronal dentin from caries-free third molars, whereas in clinical practice cavity disinfectants are applied to surfaces that include varying proportions of caries-affected dentin. Caries-affected dentin differs substantially from sound dentin in terms of mineral density, collagen cross-link integrity, and tubule patency, all of which may modify the response to disinfectant treatment and subsequent bonding [25]. Consequently, the bond strength values and group rankings reported here may not directly translate to clinical bonding on caries-affected substrates, and caution should be exercised in extrapolating these findings to routine restorative scenarios. Second, thermocycling at 10,000 cycles simulates approximately one year of intraoral aging under controlled conditions, but cannot fully replicate the complexity of the oral environment, including the combined effects of masticatory loading, enzymatic activity from saliva, and biofilm-associated acid challenge. Third, the study evaluated a single universal adhesive in self-etch mode; the interaction effects observed between disinfectant type and bond durability may differ with other adhesive formulations or etching strategies. Therefore, the clinical applicability of the present findings is limited to MDP-containing universal adhesives used in self-etch mode, and the disinfectant-adhesive interactions observed here should not be generalized to etch-and-rinse protocols or adhesive systems with different monomer compositions. Finally, the absence of SEM or nano-CT analysis of the adhesive interfaces limits mechanistic interpretation of the observed µTBS differences, and future work incorporating interfacial characterization would strengthen the conclusions drawn here.

## 5. Conclusions

Within the limitations of this in vitro study, the following conclusions were drawn:

1. The type of cavity disinfectant significantly influenced the µTBS of the 10-MDP-containing universal adhesive in self-etch mode. In particular, 5.25% NaOCl produced the lowest immediate µTBS, and 2% CHX also yielded significantly reduced 24 h values compared to the control, HOCl, and CPB groups—with NaOCl and CHX being statistically indistinguishable from each other at baseline. In contrast, 200 ppm HOCl and the MDPB-containing Clearfil SE Protect system produced 24 h values statistically comparable to the untreated control.

2. Thermocycling (10,000 cycles) significantly reduced µTBS in the control, CPB, and NaOCl groups. CHX demonstrated notable stability (27.1 to 27.2 MPa, *p* = 0.960), while HOCl showed a numerically substantial but statistically non-significant reduction (36.1 to 30.9 MPa, *p* = 0.086), highlighting the divergent aging behavior of these two agents.

3. Among the disinfectant-treated groups, the Clearfil SE Protect system demonstrated the most favorable combination of immediate and thermocycled bond strength, suggesting that integrated antibacterial adhesive systems may represent a promising alternative to conventional separate disinfection steps.

4. The NaOCl-treated group showed the highest proportion of adhesive failure modes after thermocycling, reflecting the most compromised interfacial integrity, and exhibited the greatest relative decrease in µTBS among all groups. HOCl produced acceptable immediate bond values statistically comparable to the untreated control but showed a numerically substantial, though statistically non-significant, reduction after aging, indicating that further optimization of its application protocol is needed before clinical recommendation alongside MDP-based universal adhesives.

## Figures and Tables

**Table 1:** Experimental groups and cavity disinfection protocols.

Group	Disinfection Protocol	Adhesive System	Application Details
Control	No disinfectant applied	Clearfil S3 Bond Universal (Kuraray Noritake, Tokyo, Japan)	Self-etch mode according to manufacturer’s instructions
Clearfil Protect Bond (Kuraray Noritake, Tokyo, Japan)	Clearfil SE Protect system (MDPB-containing primer)	Clearfil SE Protect (Kuraray Noritake, Tokyo, Japan)	Two-step self-etch antibacterial adhesive system applied according to manufacturer’s instructions
Chlorhexidine (Consepsis, Ultradent Products, South Jordan, UT, USA)	2% chlorhexidine digluconate	Clearfil S3 Bond Universal	Applied for 60 s, gently air-dried before adhesive application
NaOCl (Chloraxid; Cerkamed, Skoczów, Poland)	5.25% sodium hypochlorite	Clearfil S3 Bond Universal	Applied for 30 s, rinsed with water for 30 s and gently air-dried before adhesive application
HOCl (Superox; Ankara, Turkey)	200 ppm hypochlorous acid solution	Clearfil S3 Bond Universal	Applied for 30 s, excess solution was removed by blot-drying with a pellet, leaving the surface moist before adhesive application

HOCl, hypochlorous acid; NaOCl, sodium hypochlorite; MDPB, methacryloyloxydodecylpyridinium bromide. All bonding procedures were performed using the self-etch mode of the universal adhesive according to the manufacturer’s instructions.

**Table 2:** Mean microtensile bond strength values (MPa) with standard deviations.

Group	Immediate	After TC	*p*-Value
Control	39.7 (3.3) ^A^	31.9 (3.7) ^a^	0.001
Clearfil Protect Bond	34.3 (2.0) ^B^	28.5 (2.9) ^a^	0.037
Chlorhexidine	27.1 (2.2) ^C^	27.2 (3.5) ^a^	0.960
HOCl	36.1 (1.8) ^AB^	30.9 (2.0) ^a^	0.086
NaOCl	23.1 (3.5) ^C^	17.7 (3.3) ^b^	0.002

HOCl, hypochlorous acid; NaOCl, sodium hypochlorite; TC, thermocycling. Values are presented as mean (standard deviation) in MPa. The number of teeth per group per time point (*n* = 6). Uppercase letters indicate significant differences among groups at the immediate evaluation, and lowercase letters indicate significant differences among groups after thermocycling (Tukey HSD post hoc test, *p* < 0.05). Overall effects of disinfectant group, aging condition, and their interaction were analyzed using analysis of variance (ANOVA).

**Table 3:** Distribution of failure modes (%).

Group	Time	*n*	Adhesive (%)	Mixed (%)	Cohesive Dentin (%)	Cohesive Composite (%)
Control	Immediate	58	10.3	72.4	15.5	1.7
	After TC	65	36.9 *	52.3	9.2	1.5
Clearfil Protect Bond	Immediate	57	31.6	45.6	14.0	8.8
	After TC	60	38.3	46.7	11.7	3.3
Chlorhexidine	Immediate	62	38.7	41.9	16.1	3.2
	After TC	61	39.3	41.0	14.8	4.9
HOCl	Immediate	58	17.2	56.9	20.7	5.2
	After TC	54	42.6 *	46.3	11.1	0.0
NaOCl	Immediate	57	52.6	35.1	12.3	0.0
	After TC	62	61.3	27.4	8.1	3.2

HOCl, hypochlorous acid; NaOCl, sodium hypochlorite; TC, thermocycling. *n* = number of fractured specimens evaluated for failure mode analysis. The number of beams varied slightly among groups due to specimen trimming and pre-test handling procedures. * Statistically significant increase in adhesive failure compared to immediate values within the same group (Fisher’s exact test, *p* < 0.05).

## Data Availability

The data that support the findings of this study are available on request from the corresponding authors. The data are not publicly available due to privacy or ethical restrictions.

## References

[1] Ricucci D., Siqueira J.F., Rôças I.N., Lipski M., Shiban A., Tay F.R. (2020). Pulp and dentine responses to selective caries excavation: A histological and histobacteriological human study. J. Dent..

[2] Elgezawi M., Haridy R., Abdalla M.A., Heck K., Draenert M., Kaisarly D. (2022). Current strategies to control recurrent and residual caries with resin composite restorations: Operator- and material-related factors. J. Clin. Med..

[3] Tjäderhane L., Nascimento F.D., Breschi L., Mazzoni A., Tersariol I.L., Geraldeli S., Tezvergil-Mutluay A., Carrilho M.R., Carvalho R.M., Tay F.R. (2013). Optimizing dentin bond durability: Control of collagen degradation by matrix metalloproteinases and cysteine cathepsins. Dent. Mater..

[4] Carrilho M.R., Carvalho R.M., de Goes M.F., di Hipólito V., Geraldeli S., Tay F.R., Pashley D.H., Tjäderhane L. (2007). Chlorhexidine preserves dentin bond in vitro. J. Dent. Res..

[5] Carrilho M.R., Geraldeli S., Tay F., de Goes M.F., Carvalho R.M., Tjäderhane L., Reis A.F., Hebling J., Mazzoni A., Breschi L. (2007). In vivo preservation of the hybrid layer by chlorhexidine. J. Dent. Res..

[6] Kiuru O., Sinervo J., Vähänikkilä H., Anttonen V., Tjäderhane L. (2021). Mmp inhibitors and dentin bonding: Systematic review and meta-analysis. Int. J. Dent..

[7] Breschi L., Maravic T., Comba A., Cunha S.R., Loguercio A.D., Reis A., Hass V., Cadenaro M., Mancuso E., Mayer-Santos E. (2020). Chlorhexidine preserves the hybrid layer in vitro after 10-years aging. Dent. Mater..

[8] Abuhaimed T.S., Neel E.A.A. (2017). Sodium hypochlorite irrigation and its effect on bond strength to dentin. Biomed. Res. Int..

[9] Zhou W., Feng S., Chu X., Xu S., Zeng X. (2025). Effect of collagen crosslinkers on sodium hypochlorite treated dentin bond strength: A systematic review and meta-analysis. Front. Bioeng. Biotechnol..

[10] Zhou L., Wang Y., Yang H., Guo J., Tay F.R., Huang C. (2015). Effect of chemical interaction on the bonding strengths of self-etching adhesives to deproteinised dentine. J. Dent..

[11] Tsai C.F., Chung J.J., Ding S.J., Chen C.C. (2024). In vitro cytotoxicity and antibacterial activity of hypochlorous acid antimicrobial agent. J. Dent. Sci..

[12] Block M.S., Rowan B.G. (2020). Hypochlorous acid: A review. J. Oral Maxillofac. Surg..

[13] Kunawarote S., Nakajima M., Shida K., Kitasako Y., Foxton R.M., Tagami J. (2010). Effect of dentin pretreatment with mild acidic HOCl solution on microtensile bond strength and surface pH. J. Dent..

[14] Sanon K., Tichy A., Thanatvarakorn O., Prasansuttiporn T., Yonekura K., Hosaka K., Otsuki M., Nakajima M. (2022). Application of sulfinate agent in conjunction with HOCl smear-layer deproteinization improves dentin bonding durability of one-step self-etch adhesives. J. Adhes. Dent..

[15] Imazato S., Kuramoto A., Takahashi Y., Ebisu S., Peters M.C. (2006). In vitro antibacterial effects of the dentin primer of Clearfil Protect Bond. Dent. Mater..

[16] Muratovska I., Kitagawa H., Hirose N., Kitagawa R., Imazato S. (2018). Antibacterial activity and dentin bonding ability of combined use of Clearfil SE Protect and sodium hypochlorite. Dent. Mater. J..

[17] Wang R., Shi Y., Li T., Pan Y., Cui Y., Xia W. (2017). Adhesive interfacial characteristics and the related bonding performance of four self-etching adhesives with different functional monomers applied to dentin. J. Dent..

[18] Carrilho E., Cardoso M., Ferreira M.M., Marto C.M., Paula A., Coelho A.S. (2019). 10-MDP based dental adhesives: Adhesive interface characterization and adhesive stability-a systematic review. Materials.

[19] Kazemi-Yazdi H., Saeed-Nezhad M., Rezaei S. (2020). Effect of Chlorhexidine on durability of two self-etch adhesive systems. J. Clin. Exp. Dent..

[20] Shen J., Xie H., Wang Q., Wu X., Yang J., Chen C. (2020). Evaluation of the interaction of chlorhexidine and MDP and its effects on the durability of dentin bonding. Dent. Mater..

[21] Van Meerbeek B., Yoshihara K., Yoshida Y., Mine A., De Munck J., Van Landuyt K.L. (2011). State of the art of self-etch adhesives. Dent. Mater..

[22] Grazioli G., de León Cáceres E., Tessore R., Lund R.G., Monjarás-Ávila A.J., Lukomska-Szymanska M., Hardan L., Bourgi R., Cuevas-Suárez C.E. (2024). In vitro bond strength of dentin treated with sodium hypochlorite: Effects of antioxidant solutions. Antioxidants.

[23] Gascón R., Forner L., Llena C. (2023). The effect of antioxidants on dentin bond strength after application of common endodontic irrigants: A systematic review. Materials.

[24] Sanon K., Hatayama T., Tichy A., Thanatvarakorn O., Prasansuttiporn T., Wada T., Ikeda M., Hosaka K., Nakajima M. (2022). Smear layer deproteinization with NaOCl and HOCl: Do application/wash-out times affect dentin bonding of one-step self-etch adhesives. Dent. Mater. J..

[25] de Almeida Neves A., Coutinho E., Cardoso M.V., Lambrechts P., Van Meerbeek B. (2011). Current concepts and techniques for caries excavation and adhesion to residual dentin. J. Adhes. Dent..

